# A Care Process Model to Deliver ^177^Lu-Dotatate Peptide Receptor Radionuclide Therapy for Patients With Neuroendocrine Tumors

**DOI:** 10.3389/fonc.2018.00663

**Published:** 2019-01-09

**Authors:** Pashtoon Murtaza Kasi, Catherine L. Maige, Faisal Shahjehan, Jessica M. Rodgers, Debora L. Aloszka, Ashton Ritter, Margaret L. Andrus, Jessica M. Mcmillan, Kabir Mody, Akash Sharma, Manoj K. Jain

**Affiliations:** ^1^Division of Oncology, Department of Internal Medicine, Mayo Clinic, Jacksonville, FL, United States; ^2^Division of Nuclear Medicine, Department of Radiology, Mayo Clinic, Jacksonville, FL, United States

**Keywords:** peptide receptor radionuclide therapy, PRRT, ^177^Lu-Dotatate, neuroendocrine tumor, somatostatin receptor, NET, lutathera, theranostics

## Abstract

**Purpose:** To develop a care process model for the delivery of peptide receptor radionuclide therapy (PRRT) with lutetium-177 (^177^Lu)-Dotatate for the treatment of somatostatin receptor-positive gastroenteropancreatic neuroendocrine tumors (GEP-NETs).

**Methods:** A multidisciplinary, structured PRRT process model was established. Over the last 9 months, meetings were held bi-weekly to discuss the logistics of clinical trials. Meetings are still held regularly at the Mayo Clinic Florida to discuss plans regarding commercially available PRRT treatments. The process model has evolved as we have treated patients on both clinical trials and commercial treatments.

**Results:** An effective process model was formulated. We had 5 patients on our Expanded Access Program (EAP) clinical trial. Our ability to be a part of the EAP allowed us to understand the mechanics of how to treat these patients, and what was involved before it became commercially available. Since commercial availability of the ^177^Lu-Dotatate, more than 50 treatments (>20 patients) have already been completed, with several new patients getting started on treatment every week. Our nuclear medicine department receives continual requests to schedule new patients for PRRT. This can be attributed to our streamlined approach in delivering PRRT to our patients.

**Conclusion:** A thorough procedural approach was formulated to provide patients with PRRT. Experiences and challenges led to refinement, which has allowed the process to advance. This development could lead to better patient outcomes, treatment efficiency, and a reference standard for other institutions trying to develop this at their location.

## Introduction

**Figure 1:**
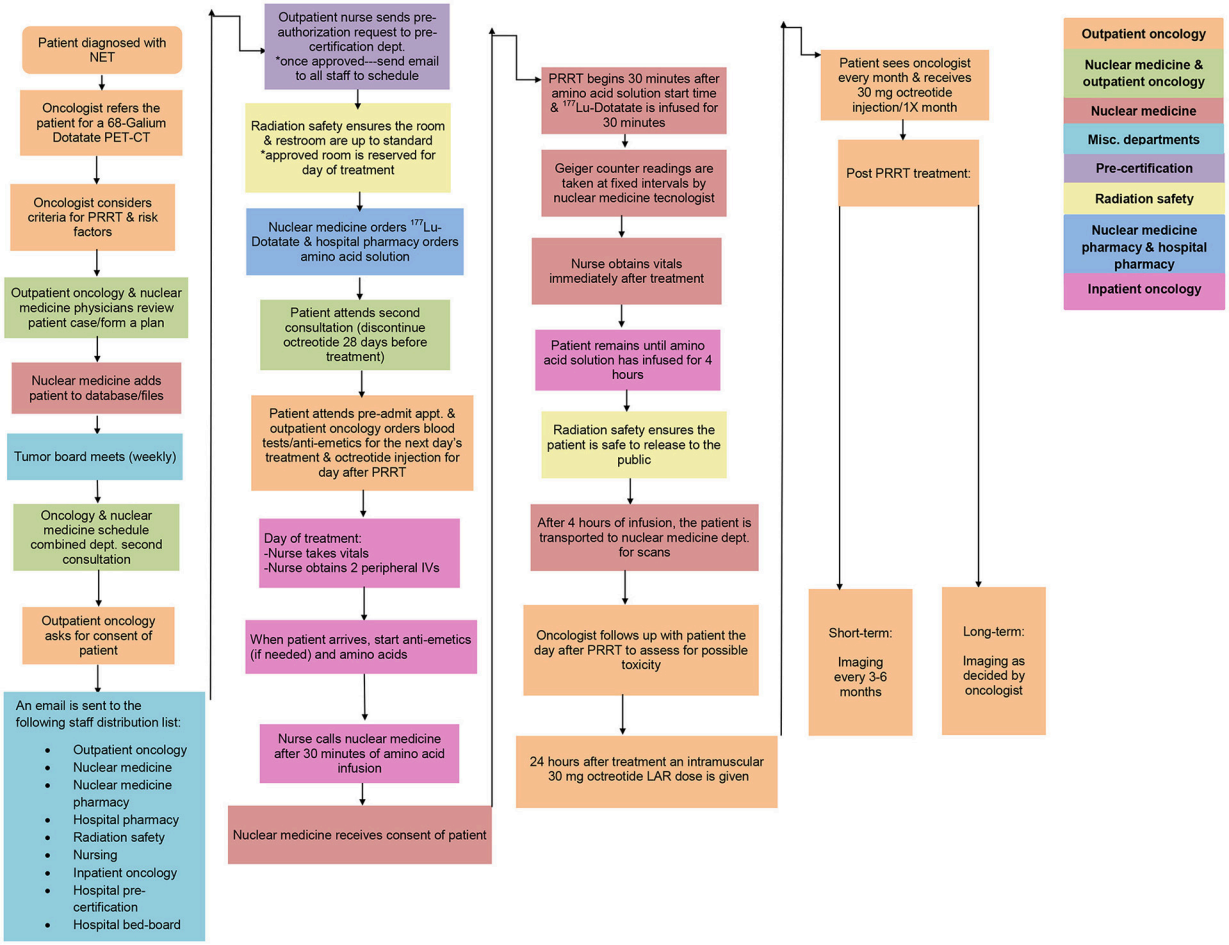
Summary of the work-flow/process model for delivering PRRT at our institution. The process was refined over many PDSA cycles. It can be adopted and/or refined as per the needs of the institution.

**Figure 2:**
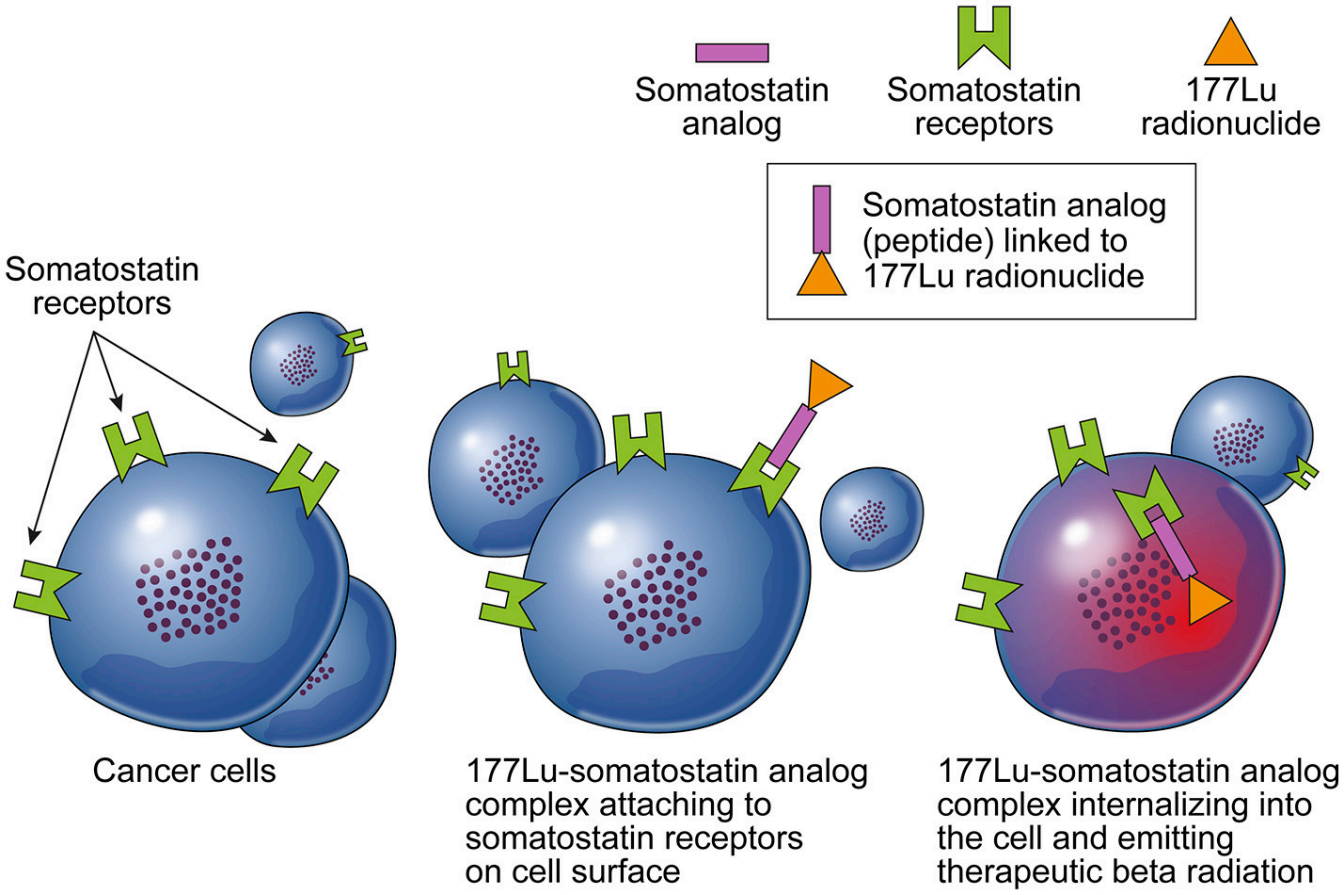
Simplistic overview illustrating the ^177^Lu-Dotatate peptide receptor radionuclide therapy - PRRT for somatostatin-receptor-positive neuroendocrine tumors. Illustration: (Steven D. Orwoll – Mayo Clinic Media).

On January 26, 2018, the Food and Drug Administration (FDA) approved peptide receptor radionuclide therapy (PRRT) with 177 Lu-Dotatate for the treatment of somatostatin receptor-positive gastroenteropancreatic neuroendocrine tumors (GEP-NETs) (1). These neuroendocrine tumors (NETs) are derived from the foregut (esophagus, stomach, pancreas, duodenum), midgut (appendix, ileum, cecum, ascending colon), and hindgut (distal large bowel and rectum); only the treatment of gastrointestinal NETs are approved. The aim of this project is to report on the creation and ongoing evolution of a process model for PRRT (Figures 1, 2); this model is for cancer centers to provide PRRT for their patients, who may otherwise have limited options. This process model was developed based on methodology described in key clinical trials leading to the approval of PRRT. We adopted a “plan, do, study, act (PDSA) strategy”, which allowed the process model to continuously develop (see Supplementary Figure 2). This novel treatment involves much orchestration and a multidisciplinary approach to the treatment including, but not limited to the following departments: medical oncology, nuclear medicine, nuclear medicine pharmacy, hospital pharmacy, radiation safety, nursing, inpatient oncology, hospital pre-certification, and the hospital bed-board. This proposed process model can serve as a model for other institutions going forward.

## Methods

The following key stages were identified:

### Diagnosis

A patient is diagnosed with a neuroendocrine tumor of the gastrointestinal tract (foregut, midgut, or hindgut). An oncologist refers the patient for a 68-Gallium Dotatate PET-CT or an Octreoscan. This allows the oncologist to confirm the presence of somatostatin positive receptors on the tumor (2). Illustrations that are available online and some created by us internally e.g., Figure 2 help explain the treatment to the patient.

### Inclusion/Exclusion Criteria

The patient must have the following criteria: NET (metastasized or “locally advanced”), inoperable tumor, histologically confirmed, disease progression when on octreotide treatments, Karnofsky Performance Status score of at least 60, adequate bone marrow function sufficient renal and hepatic function as per NCCN guidelines (Version 3:2018), well-differentiated tumor (Ki67 index of 20% or less), cytotoxic chemotherapy and/or radiotherapy > 1 month before PRRT, lanreotide/octreotide treatments (> 4 weeks from long-acting preparations or 72 h from short-acting ones) (3, 4). The following factors must be considered in addition to patient's age and comorbidities: prior locoregional therapies, prior therapies to the liver and trans-arterial chemoembolization (TACE, TAE) (5).

### Coordination With Nuclear Medicine

The oncologist coordinates with a team of nuclear medicine physicians to review the patient's case and to proceed with the planning process. The nuclear medicine department adds the patient's case to their file database. A form with key variables (Supplementary Figure 1) was created to keep track of pertinent information and record of PRRT treatments. A tumor board is held with various physicians (must include oncologists and nuclear medicine physicians) to assess the proposed treatment plan for the specific patient. We have been able to do this at our weekly tumor board conferences.

### Pre-certification

The procedure is explained to the patient and an informed consent is obtained. If the patient agrees to PRRT treatment, then an email must be sent to the staff team listed in Figure 1. An email distribution list is helpful in keeping all the stakeholders aware of the process. The tentative start date is 4–8 weeks depending on the patient's last somatostatin analog treatment. An oncology outpatient nurse sends a pre-authorization request via inbox messaging to the pre-authorization and billing departments. Information should be obtained regarding the cost, copay, and coverage under the patient's plan. When the pre-authorization request is approved, the pre-authorization department replies to the initial email to begin the scheduling process. The outpatient oncology nurse follows this up with a communication to all the staff on the communication list with a list of dates and times in order to determine the best time for treatment. Radiation safety ensures there is a radiation safe inpatient room reserved for PRRT treatment (including a leaded restroom within the room). An approved inpatient room is reserved for the day of treatment.

### Second Consultation

The patient attends his or her second consultation appointment with medical oncology, a nurse navigator, and a nuclear medicine physician. This appointment is meant to further educate the patient on PRRT, possible side effects, and to answer questions and concerns. It is very important to consolidate this appointment with both medical oncology and nuclear medicine in order to eliminate confusion and miscommunication. Medical oncology verifies with the patient that he or she will not be on octreotide for at least 28 days prior to treatment.

### ^177^Lu-Dotatate Radiopharmaceutical

The nuclear medicine pharmacy orders the correct dosage of ^177^Lu-Dotatate. The nuclear medicine technicians set-up an efficient way of organizing the 100 mL saline bag, lead can of ^177^Lu-Dotatate, and radioactivity meter. ^*^Note: there is a 2-week lead time on ordering the dosage and is often only delivered on certain weekdays. The hospital pharmacist orders an amino acid solution for renal protection (L-Arginine 2.5% /L-Lysine 2.5% in 1000 mL 0.9% NaCl).

### Day Before Treatment

The oncologist assesses the patient. Standard blood tests are ordered, and if the patient is female and < 55 years old, a pregnancy test is also ordered. The oncologist orders anti-emetics or pre-medications as supportive care for the treatment day.

### Day of Treatment

The nurse should obtain vital signs before treatment. The nurse obtains 2 peripheral IVs. When the patient arrives, start anti-emetics, and amino acid solution. After about 30 min-1 h of infusion, call nuclear medicine. The ^177^Lu-Dotatate is administered in doses of 7.4 GBq (200 mCi) per treatment for each treatment. Nuclear medicine radiologist receives consents from the patient to begin before each treatment. Ensure PRRT treatment begins at least 30 min after the amino acid solution has started. At first, the ^177^Lu-Dotatate IV infusion is started at a slower rate in order to assess the patient and how it may affect him or her to make sure that the patient is asymptomatic. Nuclear medicine physician is consulted at regular intervals and is present throughout the procedure. If the patient is feeling well, the ^177^Lu-Dotatate IV rate is increased. If the patient begins to feel nauseous or uncomfortable, the nuclear medicine technologists may slow the rate of the infusion. All the treatment decisions are taken by nuclear medicine physician. Geiger counter readings are taken by the nuclear medicine technologist at fixed intervals. The treatment is usually completed within 30 min. The nurse should obtain vital signs immediately after the infusion is completed. The patient remains in the inpatient room as the amino acid solution must infuse for a total of 4 h. A staff member from the radiation safety team must take measurements to ensure that the patient will be safe to “release to the public.”

### Post-treatment

The second PRRT treatment is scheduled 8 weeks from the initial treatment. As an outpatient, four to twenty-four hours after the treatment, the patient returns for an intramuscular 30 mg dose of octreotide LAR. The patient should receive treatment every 8 weeks for a total of 4 treatments. The patient continues to see his or her oncologist 1 month after PRRT along with monthly intramuscular injections of 30 mg of octreotide LAR (3). Dose modifications and adjustments for future treatments are ordered as per the initial clinical trials and available manufacturer guidelines. Every 3–6 months, the oncologist orders an imaging study (CT, MRI or 68-Gallium Dotatate PET-CT). The oncologist subsequently follows up with the patient as per standard of care.

## Results

We established an in-depth method of how to provide patients with PRRT. The process model is broken into nine key stages. Figure 1 shows a comprehensive illustration of our most recent iteration. We had 5 patients on our EAP clinical trial (6). Two patients, however, withdrew from the clinical trial (1 due to disease progression after 2 treatments and 1 due to personal patient preference). Since the commercial approval, more than 50 treatments (>20 patients) have been completed, with several new patients starting treatment every week. Meetings continue to be held to review the protocol as there is an influx of patients needing commercial treatments.

## Discussion

We have developed an efficient mechanism to streamline the process of treating patients who need PRRT. This model's foundation is built upon the EAP clinical trial and our continuous learning. We established a thorough way to streamline the care of the patient, collaborate with various departments, and to determine the best path for the patient's care. The use of the PDSA approach allowed us to modify the process according to the feedback of all staff involved.

Of note, the treatment day is a hospital-based outpatient admission and all the treatment is done at one place. At our institution, the patient's care is under our hematology/oncology inpatient service with our inpatient team and oncology fellow coordinating the treatment between primary oncologist and nuclear medicine department. The patient is not transported anywhere and remains in the same treatment room till discharge. Nuclear medicine department is informed of the admission and when the amino acid solution is started. The faculty and their team come to see the patient and administer the PRRT treatment in the same room. Medical oncology team remains available for any as needed management needs and is responsible for the discharge post completion of amino acid solution. The whole process takes between 5 and 7 h. Post discharge as per the clinical trial, the long acting monthly SSA is continued. Routinely, long acting octreotide is used at our center for somatostatin receptor-positive GEP-NETs. However, lanreotide is also listed as an alternative as per NCCN (National Comprehensive Cancer Network) guidelines.

The project, although, had a few challenges. One major hurdle encountered was the amino acid solution originally prescribed for patients. The first amino acids used were alcohol based. The alcohol is very emetogenic. If the only amino acid solution available is of this kind, we recommend aggressively pre-medicating the patient with grade 5 anti-emetics. Steroids are not recommended as an anti-emetic due to its down-regulating effects on somatostatin receptor. The newer formulations do not require anti-emetics and are well-tolerated.

Another refinement in the model was the incorporation of nuclear medicine with outpatient oncology during the second consultation. This led to more effective communication and better patient education.

A new nursing position was also added as a result of this project. Our gastrointestinal oncology department now has a nurse navigator who is responsible for facilitating the patient's PRRT journey. The nurse navigator's key role is to orchestrate with the patient and the various departments involved. We continue to learn as new advancements are made. This model can be used as presented or can be adjusted to the needs of the institution.

## Author Contributions

The care process model as noted was made possible by all the departments as noted including but not limited to oncology, nuclear medicine, pharmacy, and radiation oncology. PMK and CLM wrote the initial draft. AS and MKJ provided critical feedback and input. The manuscript was then edited and finalized based on contributions from all the authors. All authors approved the final draft for publication.

### Conflict of Interest Statement

The authors declare that the research was conducted in the absence of any commercial or financial relationships that could be construed as a potential conflict of interest.
