# Supplementary figures and images for: A Care Process Model to Deliver 177Lu-Dotatate Peptide Receptor Radionuclide Therapy for Patients With Neuroendocrine Tumors
Source: Front Oncol. 2019 Jan 9;8:663. doi: 10.3389/fonc.2018.00663 (PMC6333662; doi:10.3389/fonc.2018.00663)

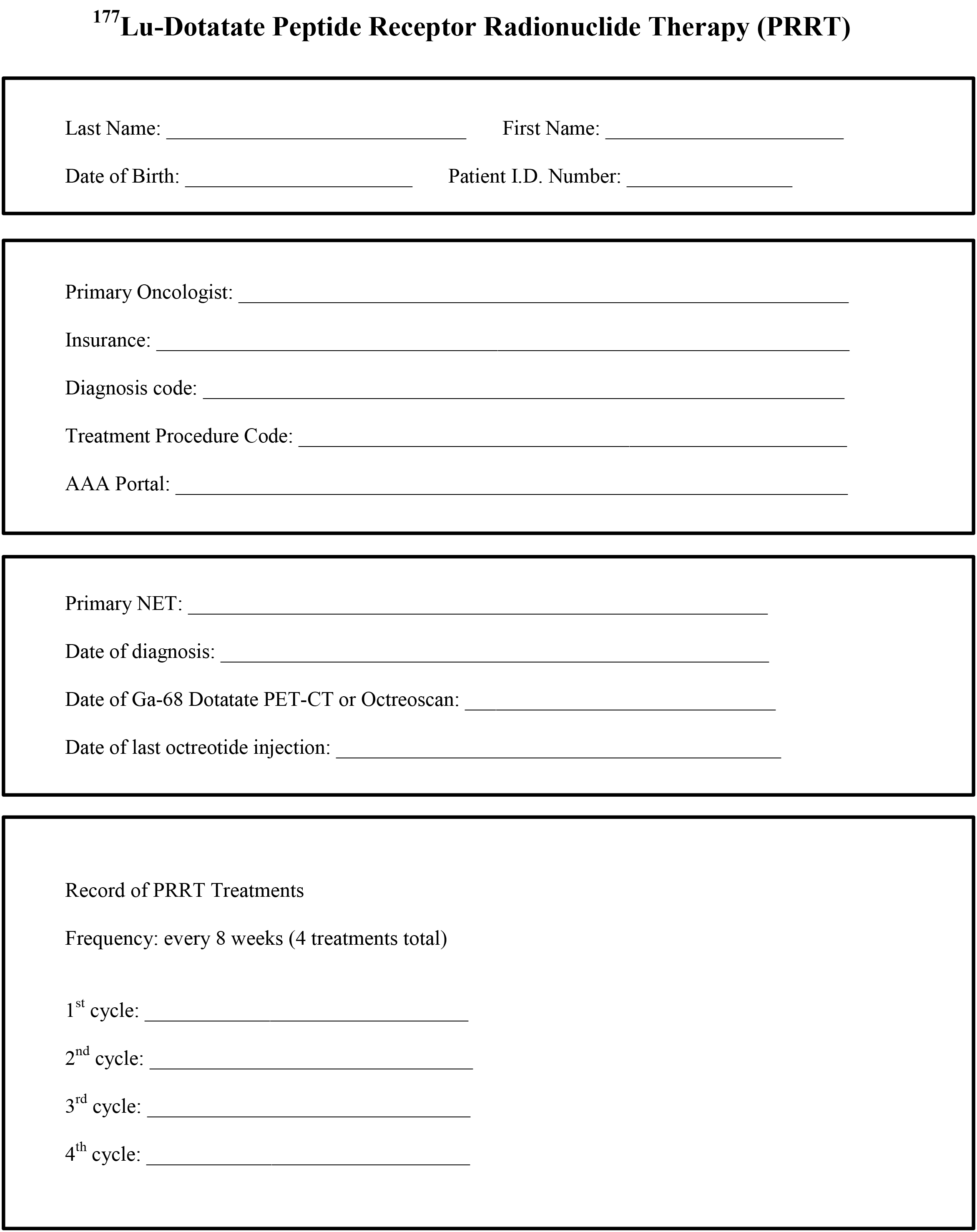

Supplement: Supplementary file 1 [file Image_1.TIF]

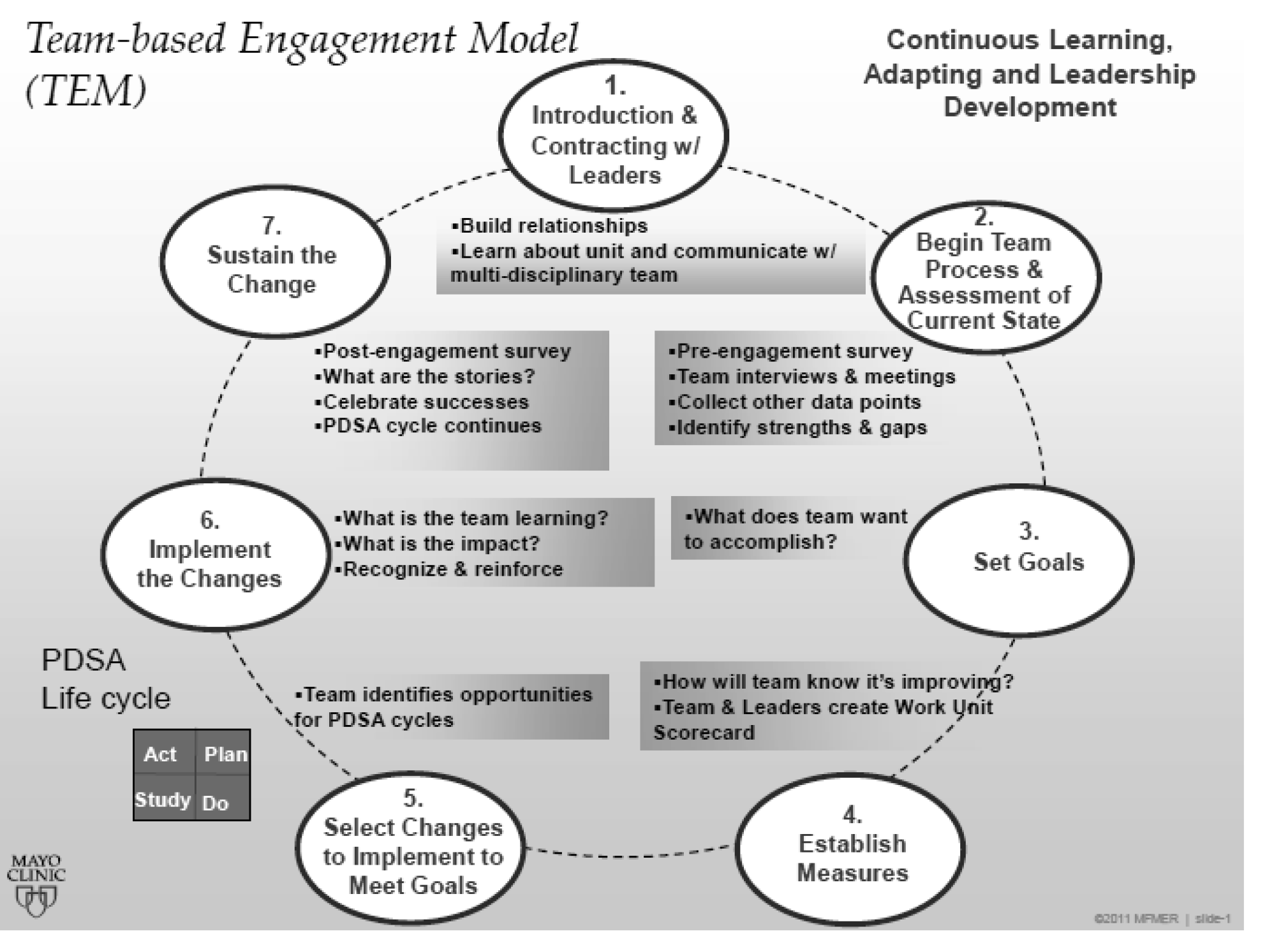

Supplement: Supplementary file 2 [file Image_2.TIF]
